# Memprot.GPCR-ModSim: modelling and simulation of membrane proteins in a nutshell

**DOI:** 10.1093/bioinformatics/btae662

**Published:** 2024-11-06

**Authors:** Remco L van den Broek, Xabier Bello, Rebecca V Küpper, Gerard J P van Westen, Willem Jespers, Hugo Gutiérrez-de-Terán

**Affiliations:** Department of Cell and Molecular Biology, Uppsala University, BMC - Box 596, Uppsala, SE 751 24, Sweden; Division of Medicinal Chemistry, Leiden Academic Centre for Drug Research, Leiden University, Leiden 2333 CC, The Netherlands; Institute of Forensic Sciences, Faculty of Medicine, University of Santiago de Compostela, Hospital Clínico Universitario de Santiago de Compostela (SERGAS) Santiago de Compostela, Galicia, 15706, Spain; Department of Cell and Molecular Biology, Uppsala University, BMC - Box 596, Uppsala, SE 751 24, Sweden; Division of Medicinal Chemistry, Leiden Academic Centre for Drug Research, Leiden University, Leiden 2333 CC, The Netherlands; Division of Medicinal Chemistry, Leiden Academic Centre for Drug Research, Leiden University, Leiden 2333 CC, The Netherlands; MOD4SIM Pharma AB, Box 2022, Uppsala, SE 750 02, Sweden; Department of Cell and Molecular Biology, Uppsala University, BMC - Box 596, Uppsala, SE 751 24, Sweden; MOD4SIM Pharma AB, Box 2022, Uppsala, SE 750 02, Sweden

## Abstract

**Summary:**

Memprot.GPCR-ModSim leverages our previous web-based protocol, which was limited to class-A G protein-coupled receptors, to become the first one-stop web server for the modelling and simulation of any membrane protein system. Motivated by the exponential growth of experimental structures and the breakthrough of deep-learning-based structural modelling, the server accepts as input either a membrane-protein sequence, in which case it reports the associated AlphaFold model, or a 3D (experimental, modelled) structure, including quaternary complexes with associated proteins and/or ligands of any kind. In both cases, the molecular dynamics (MD) protocol produces a membrane-embedded, solvated, and equilibrated system, ready to be used as a starting point for further MD simulations, including ligand-binding free energy calculations.

**Availability and implementation:**

Memprot.GPCR-ModSim web server is publicly available at https://memprot.gpcr-modsim.org/. The standalone modules for 3D modelling (PyModSim) or membrane embedding and MD equilibration (PyMemDyn) are available under CC BY-NC 4.0 license terms at the GitHub repository https://github.com/GPCR-ModSim/.

## 1 Introduction

Structural biology of membrane proteins has been revolutionized with receptor stabilization techniques (Tate and Schertler 2009), crystallization in membrane phases (Cherezov 2011), and later by developments of cryo-EM, all of which resulted in atomic-resolution structures of membrane-protein complexes (García-Nafría and Tate 2019). This growing pool of experimental structures is nowadays complemented by highly accurate structural prediction of proteins, including the remaining membrane-protein systems, through deep-learning algorithms (Jumper *et al.* 2021). However, further investigations on the structure, dynamics, and modulation of membrane proteins are challenged by the lack of rapid and effortless methods to generate membrane-embedded equilibrated protein systems.

Inspired by the first crystal structures of G protein-coupled receptors (GPCRs), we initially developed GPCR-ModSim as a web server that implemented specifically designed solutions for homology modelling and molecular dynamics (MD) simulations of class-A GPCRs (Rodríguez *et al.* 2012), arguably the most important pharmaceutical targets among all membrane proteins (Hauser *et al.* 2017). The server was designed as a modular, stepwise protocol that combined the advantages of a fully automated pipeline while allowing the advanced user to modify the recommended parameters at each stage. The protocol included several checkpoints for input/output, in a way that each module (i.e. homology modelling or MD simulations) could be used independently. Here, the PyMemDyn module within GPCR-ModSim (Gutiérrez-de-Terán *et al.* 2013) performed the necessary insertion of the receptor into a solvated model of the cellular membrane, followed by a carefully designed MD equilibration protocol. The resulting solvated receptor-membrane equilibrated complex could be used to further investigate the dynamic processes of GPCRs (Wei *et al.* 2022). GPCR-ModSim performed indeed as the best automated web-based environment in recreating target structures in the GPCR Dock 2013 competition (Kufareva *et al.* 2014). An extended version of GPCR-ModSim (Esguerra *et al.* 2016) added a multi-template homology modelling functionality, as well as an implementation of the MD equilibration protocol of pair-distance restraints derived from a comprehensive analysis of class-A GPCR crystal structures (Venkatakrishnan *et al.* 2013).

While these new functionalities added flexibility and improved performance, particularly in cases where the closest template has low homology to the query sequence, the server was still limited to class-A GPCRs. This limitation is to some extent shared with other web servers dedicated to the structural modelling of GPCRs (listed in https://docs.gpcrdb.org/external_sites.html), though many were generalized to the different GPCRs classes according to the GRAFS classification system (Fredriksson *et al.* 2003). A database storing MD trajectories for GPCRs has also been recently reported (Rodríguez-Espigares *et al.* 2020). In addition, other resources exist to perform particular steps needed to model and simulate membrane proteins: the MemProtMD database provides a membrane-embedded version of every experimental membrane-protein structure available on the PDB, after a coarse-grained simulation and reversion to an atomistic version (Newport *et al.* 2019). Similarly, the OPM database provides the Orientation of (experimentally solved) Proteins in Membranes, and the associated PPM server allows similar positionings of new structures and definition of membrane boundaries (Lomize *et al.* 2012). The web server CHARMM-GUI serves as a preliminary step for building membrane–protein complexes ready for MD simulations (Jo *et al.* 2008). However, the setup of the associated membrane–protein simulations with the last versions of GROMACS, one of the most widely used MD engines, is still tedious and time-consuming (Abraham *et al.* 2015).

In this work, we present an adaptation of the framework underlying GPCR-ModSim to implement two major features for the benefit of the membrane-protein research community: (i) the inclusion of deep-learning structural modelling by adopting the AlphaFold database and modelling engine, and (ii) the leveraging of MD protocols, with a more robust membrane embedding phase adapted to include multiple chains and non-protein elements, in both cases by simply uploading them as PDB files. Both features allow the generalization of the new web server to process any membrane-protein system, the characteristics of which are presented and illustrated here with case studies of several membrane proteins including GPCRs, transporters, and ion channels, bringing together the biggest cluster of pharmaceutical targets (Santos *et al.* 2017). A tutorial is offered as online documentation, available at https://memprot.gpcr-modsim.org/tutorials/, providing a further illustration of the stepwise process based on selected examples from the above.

## 2 Methods and features

**Figure 1.**
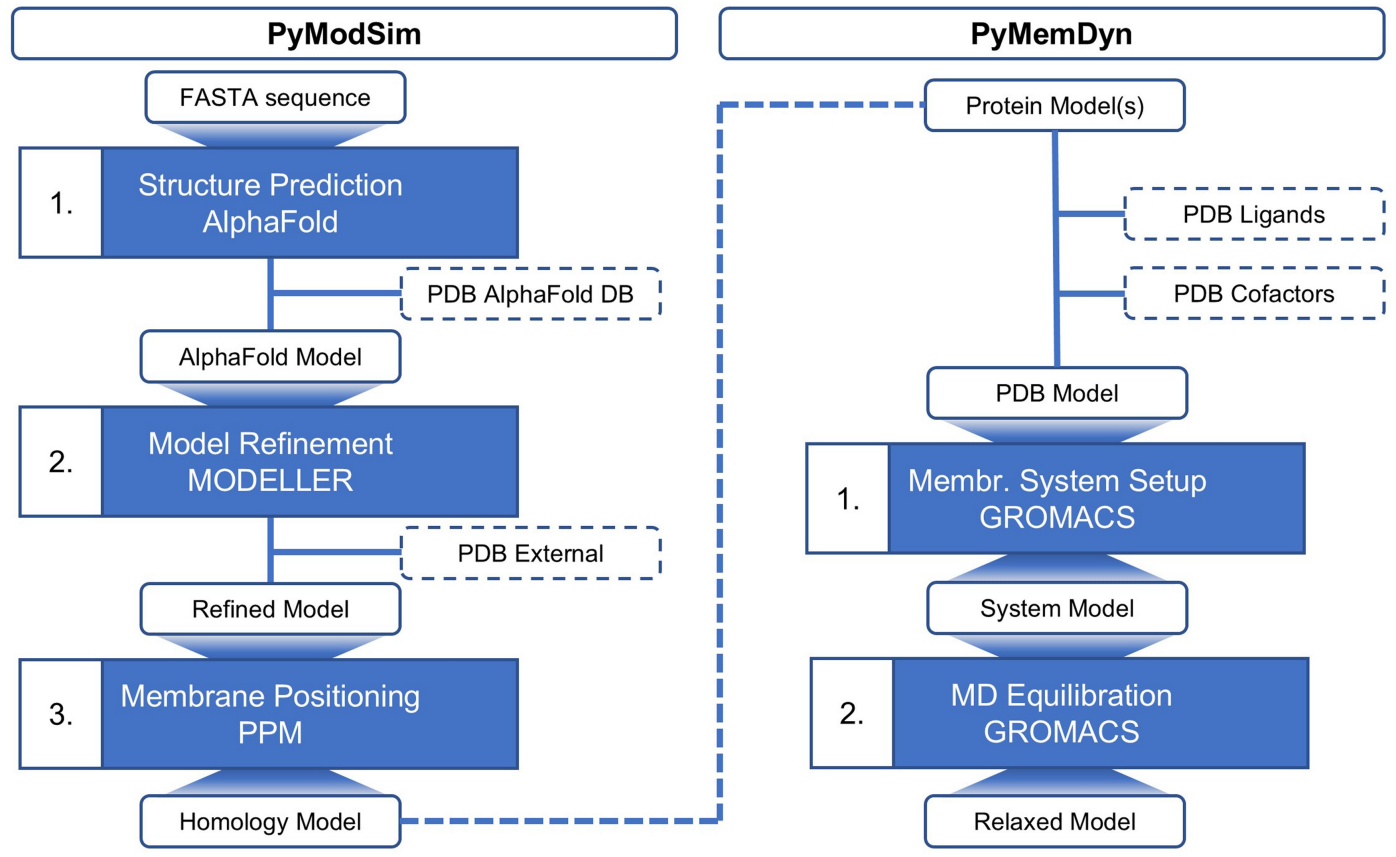
Schematic representation of the workflow within the Memprot.GPCR-ModSim web server. Each stage is depicted in blue boxes. The input and output of these stages is depicted in white boxes, with optional input indicated in dashed white boxes.

Memprot.GPCR-ModSim is a Python/Django web server connecting two modules, designed respectively for the 3D modelling (PyModSim) and MD simulation (PyMemDyn) of membrane proteins (Fig. 1). Both modules use the Python 3 programming language and communicate with several bio- and cheminformatics software (free for academics in all cases): PyModSim communicates with AlphaFold 2.0 (Jumper *et al.* 2021) for structure prediction, MODELLER 10.2 (Sali and Blundell 1993) for loop refinement, and PPM 3.0 (Lomize *et al.* 2012) for membrane positioning. PyMemDyn, in turn, communicates with LigParGen 2.1 (Dodda *et al.* 2017) for the generation of forcefield parameters, MODELLER 10.2 to fix broken loops, and GROMACS 2021 (Abraham *et al.* 2015) for the membrane embedding and MD simulation.

### 2.1 Structure prediction and refinement

The pipeline starts with the submission of a FASTA sequence, or a UniProt ID, corresponding to a membrane protein. The system initially checks if a 3D model is available in the AlphaFold database (AF-db) (Varadi *et al.* 2022), in which case it will be directly retrieved and the corresponding PDB file will be checked for potential refinement (see below), before proceeding to the next stage (membrane insertion). To speed up this process, AF-db sequences are hashed and locally stored. If the query sequence does not match a hit in the AF-db (for which a single point mutation as compared to a stored model sequence is enough), the local installation of AF2 serves as a backend to the webserver to produce the initial 3D model of the query sequence on demand.

Irrespective of its origin (i.e. AF-db or produced on demand), any model generated by AF2 might contain low-confident regions, as defined by the per-residue confidence score (pLDDT = [0,100]). Regions with pLDDT < 70 are tagged for revision in Memprot.GPCR-ModSim to ensure that long and unstructured regions do not interfere with the subsequent MD simulations. Thus, if the region corresponds to unstructured termini, these are simply removed, while unstructured loops are replaced by a polyalanine linker using MODELLER. The length of this linker is determined by the Euclidean distance between the corresponding termini of the removed region, at a ratio of one residue per two Å. The command-line version of PyModSim, available at the GitHub repository (https://github.com/GPCR-ModSim/), allows for more advanced editing options, such as modification of this ratio, the definition of the loop or termini sections to replace, or the topology, which by default assumes that the N-terminus is at the extracellular side (See Supplementary material 1).

The output of this module is offered as a downloadable bundled compressed file (model_output.tgz), containing the initial 3D model of the protein, the refined model, and a version of the latest aligned to the plane of the membrane (see Section 2.3), all of them as PDB files, together with the log file reporting the step-by-step output of the PyModSim protocol. In addition, the last 20 lines of this log file are provided on the progress window and dynamically updated, and the user can download it at any point, which is especially useful if the modelling process encountered any issue allowing the user to revise the input file and resubmit the failed job.

### 2.2 Importing a membrane protein structure

An alternative entry stage on Memprot.GPCR-ModSim is to upload an existing PDB structure of a membrane protein (Fig. 1). This can be just a single-chain protein or a multimer (typical of e.g., ion channels, GPCR oligomers, etc.). In the latter case, the user will be prompted to confirm the chain(s) that should be included in the setup of the MD simulation, indicating which transmembranal chains are used to orient the protein in the membrane (see Section 2.3). The input structure can additionally include non-protein elements (ligands, ions, structural waters, cholesterol molecules, etc.), which are recognized by the system and revised by the user, who shall specify which of these molecules should be included in the simulation and determine the class (i.e. water, ion or organic molecule) and formal charge, for further processing. Memprot.GPCR-ModSim includes a new FAQ section (https://memprot.gpcr-modsim.org/qa/) to advise novel users how to pre-process a PDB file prior uploading to Memprot.GPCR-ModSim, to fulfil the requirements of the GROMACS engine and minimize job failures.

### 2.3 Membrane embedding and parameterization of non-protein elements

Both alternative entry points (sequence input described in Section 2.1 or provided protein structure indicated in Section 2.2) converge at this stage. The protein is oriented within a planar mammalian plasma membrane positioned on the horizontal axis with our local installation of the PPM algorithm. While the original PPM server only handles the transmembranal protein chain(s) that were defined by the user, our implementation stores and applies the translation matrix to any other molecule uploaded as part of the complex, that is, associated proteins or any non-protein element, as we illustrate in the Section 3.

At this stage, and prior to the preparation of the MD simulation system, the server performs a necessary check on the input PDB file, looking for problematic regions that would cause a collapse of the MD simulation. These include missing loops, based on the detection of disconnected residues (within one chain), or missing, incomplete, or non-natural sidechains. The MODELLER routines are then called to either construct the shortest polyalanine chain that replaces the missing loop (as outlined in Section 2.1) or reconstruct the incomplete or missing sidechains.

The MD simulation system is then created and passed as input to the PyMemDyn module (see Fig. 1). This module takes care of the full parameterization of any non-protein element indicated to be retained for the MD simulation, making use of our local installation of the LigParGen engine using default parameters (Dodda *et al.* 2017), to ensure compatibility with the OPLS-AA forcefield used for the protein (Kaminski *et al.* 2001) (see Section 2.4). The full system is then embedded in a pre-equilibrated membrane model of palmitoyloleoylphosphatidylcholine using the Berger parameters (Berger *et al.* 1997), followed by the definition of a hexagonal-prism shaped box and solvation with the SPC water model, following the newest GROMACS (>5.2) routines (Abraham *et al.* 2015).

### 2.4 Relaxation protocols

The prepared system undergoes a previously optimized MD equilibration protocol, consisting of steepest-descent energy minimization followed by a partially restrained 5 ns MD simulation (Gutiérrez-de-Terán *et al.* 2013). The first 2.5 ns include a gradual relaxation of the positional restraints initially applied on all heavy atoms of protein and cofactors, (from a force constant of 1000–200 kJ mol^−1^ nm^−2^), which is followed by 2.5 ns where the softer restraints (200 kJ mol^−1^ nm^−2^) are only retained for the Cα trace of the protein(s) and heavy atoms of the cofactors. Alternatively, for class-A GPCRs, it is possible to replace the last phase by equivalent 2.5 ns MD with a set of pairwise distance restraints derived from experimental structures, which are automatically generated by taking advantage of the topological GPCR numbering (Esguerra *et al.* 2016). Additional options are available at the command-line version of PyMemDyn, available at the GitHub repository (see Supplementary material 2).

As in Section 2.1, the last 20 lines of the log file (corresponding to the ongoing MD stage) are dynamically displayed on the progress window (see Supplementary material 3), and the user can download the current PyMemDyn log file, reporting the step-by-step output of the PyMemDyn protocol, particularly useful to debug a failed job and resubmit. An example of the PyMemDyn log file is provided as Supplementary material 3. The output from MD simulations is offered as a downloadable bundled compressed file (MDoutput.tgz), containing: (i) a PyMOL script (load_gpcr.pml) to load the trajectory in the PyMOL visualization program with optimized visualization settings; (ii) the logs folder, with detailed GROMACS output from each equilibration step; (iii) the reports folder containing plots in XMGrace formatted XVG files to control that energy, volume, temperature, and pressure of the system have reached equilibrium; (iv) the log file indicating the steps performed on this module; (v) all files needed to continue a production run using GROMACS, provided along with a README.md text file with instructions. A system coming from a PyMemDyn protocol can also be easily transferred to free energy perturbation protocols, facilitating the use of, for example, ligand-binding or in silico mutagenesis through FEP simulations on membrane proteins (Vasile *et al.* 2018).

## 3 Application

To illustrate the wide applicability domain of Memprot.GPCR-ModSim, we applied the pipeline to representative proteins from diverse families of integral membrane proteins. The tutorial shows the 3D modelling capabilities illustrated with the H_3_ histamine receptor, either by retrieving the WT sequence from the AF-db (UniProt ID: Q9Y5N1), or by modelling a mutant version of the receptor from sequence with AF2. The MD simulation capabilities are illustrated on selected cases that include GPCRs from class B2 (adhesion, receptor L_1_), class C (glutamate, receptor mGLU_6_), and class F (frizzled, receptor frizzled-6), as well as solute carriers (SLC DAT_1_), and ion channels (Na channel protein type 11 subunit alpha). In all these five examples, the full pipeline from sequence to MD was executed (i.e. both PyModSim and PyMemDyn protocols, see Supplementary material 4A and B). In addition, we illustrate the capability of the PyMemDyn MD module to simulate two complex structures: The X-ray structure of the cannabinoid receptor 1 (CB_1_) bound with both orthosteric and allosteric ligands (PDB: 6KQI) represents a ligand-bound protein simulation; the second example includes a model generated by Navarro et al. of a tetramer consisting of two copies of the A_1_ adenosine receptor, heterodimerizing with two copies of the A_2A_ adenosine receptor, and in complex with both the G_i_ and the G_s_ G proteins (Navarro *et al.* 2018), showing the suitability of PyMemDyn on handling multimeric structures. The relaxed proteins are shown in Supplementary material 4C. These examples illustrate how PyModSim and PyMemDyn modules within Memprot.GPCR-ModSim can simulate diverse protein structures and protein complexes directly from sequence.

In conclusion, starting with the skeleton of a successful web-based service originally developed to model and simulate class-A GPCRs, we provide a new server that allows to model the 3D structure of any membrane protein, embed the generated structure in the membrane, and prepare and perform MD simulations of complex membrane systems. We illustrate the capabilities of Memprot.GPCR-ModSim with case studies including multimers (homo or heteromers), typical of, for example, ion channels or transporters, ternary complexes of GPCRs, and pharmacologically relevant systems that include organic molecules such as orthosteric or allosteric modulators, which are directly uploaded as structure files without the need of further edition or manipulation by the user. With the increased availability of high-quality protein structures, our automated protocols for structural modelling and MD simulations will drastically increase the accessibility of advanced computational investigations to membrane proteins.

## Supplementary Material

btae662_Supplementary_Data

## References

[btae662-B1] Abraham MJ , Murtola T, Schulz R et al GROMACS: high performance molecular simulations through Multi-Level parallelism from laptops to supercomputers. SoftwareX 2015;1-2:19–25.

[btae662-B2] Berger O , Edholm O, Jähnig F. Molecular dynamics simulations of a fluid bilayer of dipalmitoylphosphatidylcholine at full hydration, constant pressure, and constant temperature. Biophys J 1997;72:2002–13. 9129804 10.1016/S0006-3495(97)78845-3 PMC1184396

[btae662-B3] Cherezov V. Lipidic cubic phase technologies for membrane protein structural studies. Curr Opin Struct Biol 2011;21:559–66. 21775127 10.1016/j.sbi.2011.06.007 PMC3164297

[btae662-B4] Dodda LS , Cabeza De Vaca I, Tirado-Rives J et al LigParGen web server: an automatic OPLS-AA parameter generator for organic ligands. Nucleic Acids Res 2017;45:W331–6. 28444340 10.1093/nar/gkx312 PMC5793816

[btae662-B5] Esguerra M , Siretskiy A, Bello X et al GPCR-ModSim: a comprehensive web based solution for modeling G-Protein coupled receptors. Nucleic Acids Res 2016;44:W455–62. 27166369 10.1093/nar/gkw403 PMC4987938

[btae662-B6] Fredriksson R , Lagerström MC, Lundin L-G et al The G-protein-coupled receptors in the human genome form five main families. Phylogenetic analysis, paralogon groups, and fingerprints. Mol Pharmacol 2003;63:1256–72. 12761335 10.1124/mol.63.6.1256

[btae662-B7] García-Nafría J , Tate CG. Cryo-EM structures of GPCRs coupled to G_s_, G_i_ and g_o_. Mol Cell Endocrinol 2019;488:1–13. 30930094 10.1016/j.mce.2019.02.006

[btae662-B8] Gutiérrez-de-Terán H , Bello X, Rodríguez D. Characterization of the dynamic events of GPCRs by automated computational simulations. Biochem Soc Trans 2013;41:205–12. 23356284 10.1042/BST20120287

[btae662-B9] Hauser AS , Attwood MM, Rask-Andersen M et al Trends in GPCR drug discovery: new agents, targets and indications. Nat Rev Drug Discov 2017;16:829–42. 29075003 10.1038/nrd.2017.178 PMC6882681

[btae662-B10] Jo S , Kim T, Iyer VG et al CHARMM‐GUI: a web‐based graphical user interface for CHARMM. J Comput Chem 2008;29:1859–65. 18351591 10.1002/jcc.20945

[btae662-B11] Jumper J , Evans R, Pritzel A et al Highly accurate protein structure prediction with AlphaFold. Nature 2021;596:583–9. 34265844 10.1038/s41586-021-03819-2 PMC8371605

[btae662-B12] Kaminski GA , Friesner RA, Tirado-Rives J et al Evaluation and reparametrization of the OPLS-AA force field for proteins via comparison with accurate quantum chemical calculations on peptides. J Phys Chem B 2001;105:6474–87.

[btae662-B13] Kufareva I , Katritch V, Stevens RC, Participants of GPCR Dock 2013 et al Advances in GPCR modeling evaluated by the GPCR dock 2013 assessment : meeting new challenges. Structure 2014;22:1120–39. 25066135 10.1016/j.str.2014.06.012 PMC4126895

[btae662-B14] Lomize MA , Pogozheva ID, Joo H et al OPM database and PPM web server: resources for positioning of proteins in membranes. Nucleic Acids Res 2012;40:D370–76. 21890895 10.1093/nar/gkr703 PMC3245162

[btae662-B15] Navarro G , Cordomí A, Brugarolas M et al Cross-communication between G_i_ and G_s_ in a G-Protein-coupled receptor heterotetramer guided by a receptor C-Terminal domain. BMC Biol 2018;16:24–15. 29486745 10.1186/s12915-018-0491-x PMC6389107

[btae662-B16] Newport TD , Sansom MSP, Stansfeld PJ. The MemProtMD database: a resource for Membrane-Embedded protein structures and their lipid interactions. Nucleic Acids Res 2019;47:D390–D397. 30418645 10.1093/nar/gky1047 PMC6324062

[btae662-B17] Rodríguez D , Bello X, Gutiérrez-de-Terán H. Molecular modelling of G protein-coupled receptors through the web. Mol Inform 2012;31:334–41. 27477263 10.1002/minf.201100162

[btae662-B18] Rodríguez-Espigares I , Torrens-Fontanals M, Tiemann JKS et al GPCRmd uncovers the dynamics of the 3D-GPCRome. Nat Methods 2020;17:777–87. 32661425 10.1038/s41592-020-0884-y

[btae662-B19] Sali A , Blundell TL. Comparative protein modelling by satisfaction of spatial restraints. J Mol Biol 1993;234:779–815. 8254673 10.1006/jmbi.1993.1626

[btae662-B20] Santos R , Ursu O, Gaulton A et al A comprehensive map of molecular drug targets. Nat Rev Drug Discov 2017;16:19–34. 27910877 10.1038/nrd.2016.230 PMC6314433

[btae662-B21] Tate CG , Schertler GFX. Engineering G protein-coupled receptors to facilitate their structure determination. Curr Opin Struct Biol 2009;19:386–95. 19682887 10.1016/j.sbi.2009.07.004

[btae662-B22] Varadi M , Anyango S, Deshpande M et al AlphaFold protein structure database: massively expanding the structural coverage of protein-sequence space with high-accuracy models. Nucleic Acids Res 2022;50:D439–D444. 34791371 10.1093/nar/gkab1061 PMC8728224

[btae662-B23] Vasile S , Esguerra M, Jespers W et al Characterization of ligand binding to GPCRs through computational methods. Methods Mol Biol 2018;1705:23–44. 29188557 10.1007/978-1-4939-7465-8_2

[btae662-B24] Venkatakrishnan AJ , Deupi X, Lebon G et al Molecular signatures of G-protein-coupled receptors. Nature 2013;494:185–94. 23407534 10.1038/nature11896

[btae662-B25] Wei S , Thakur N, Ray AP et al Slow conformational dynamics of the human A_2A_ adenosine receptor are temporally ordered. Structure 2022;30:329–37.e5. 34895472 10.1016/j.str.2021.11.005 PMC8897252

