# Supplementary material for: Memprot.GPCR-ModSim: modelling and simulation of membrane proteins in a nutshell
Source: Bioinformatics. 2024 Nov 6;40(11):btae662. doi: 10.1093/bioinformatics/btae662 (PMC11578594; doi:10.1093/bioinformatics/btae662)
Supplement: btae662_Supplementary_Data [file btae662_supplementary_data.docx]

SI 1. **PyModSim command line options.**

usage: pymodsim [-h] [-v] [-n NSTEP] [-s SEQUENCE] [-p PDB] [-N NTERM] [-C CTERM] [-l LOOP]

[-f LOOP_FILL] [-t TOPOLOGY] [-c CHAIN]

== Create prepared homology models given a sequence. ==

options:

-h, --help show this help message and exit

-v, --version show program's version number and exit

-n NSTEP, --nstep NSTEP

PyModSim steps you wish to execute. This allowes you modify the

model preparation steps - see documentation Options: (0) Full, (1)

Homology|AlphaFold, (2) ModPrep|MODELLER, (3) Orientation|PPM, and

(23) ModPrep+Orientation

-s SEQUENCE, --seq SEQUENCE

Name of the sequence for which to create an homology model. -s is

only required if -n = 0, 1 or 2. Use the fasta extension. (example:

-s myseq.fasta)

-p PDB, --pdb PDB Name of the protein to process. -p is only required if -n = 2 or 3.

Use the pdb extension. (example: -p myprot.pdb)

-N NTERM, --Nterm NTERM

Residue number at which to cut the N-terminus. Note: the chain up

AND including the given residue will be removed. -N is only used if

-n = 2. If you wish to use the default cutoff, don't specify -N. If

you wish not to cut the N-term: set -N = 0

-C CTERM, --Cterm CTERM

Residue number at which to cut the C-terminus. Note: the chain from

AND including the given residue will be removed. -C is only used if

-n = 2. If you wish to use the default cutoff, don't specify -C. If

you wish not to cut the C-term: set -C = 0

-l LOOP, --loop LOOP Residue numbers at which to cut loop(s). Define the first and last

residue of the loop you wish to cut ('-' delimited) If there are

multiple loops to cut, delimit the loop cuts with a ',' (example: -l

101-131,230-250). If you do not with to cut any loops: set -l = 0

-f LOOP_FILL, --loop_fill LOOP_FILL

Amount of Å per AA to fill cut loops. The total distance is

calculated from the coordinates of the remaining residues. The AA

contour length is 3.4-4.0 Å, To allow for flexibility in the loop,

2.0 Å/AA (default) is suggested. (example: -f 2.0)

-t TOPOLOGY, --topology TOPOLOGY

Indicate the topology of the N-term within the protein structure.

'out': extracellular N-term (default), 'in': intracellular N-term.

-c CHAIN, --chain CHAIN

Only use if -n = 3 (i.e. only PPM). If more than 1 chain, add a

comma-seperated list of the chain identifiers. (example: -c A,B,C)

SI 2. **PyMemPyn command line options.**

usage: pymemdyn [-h] [-v] [-b OWN_DIR] [-r REPO_DIR] -p PDB [-l LIGAND]

[--lc LIGAND_CHARGE] [-w WATERS] [-i IONS]

[--full_relax FULL_RELAX] [--res RESTRAINT] [-f LOOP_FILL]

[-q QUEUE] [-d]

== Setup Molecular Dynamics for Membrane Proteins given a PDB. ==

optional arguments:

-h, --help show this help message and exit

-v, --version show program's version number and exit

-b OWN_DIR Working dir if different from actual dir

-r REPO_DIR Path to templates of fixed files. If not provided,

take the value from settings.TEMPLATES_DIR.

-p PDB Name of the PDB file to insert into membrane for MD

(mandatory). Use the .pdb extension. (e.g. -p

myprot.pdb)

-l LIGAND, --lig LIGAND

Ligand identifiers of ligands present within the PDB

file. If multiple ligands are present, give a comma-

delimited list.

--lc LIGAND_CHARGE Charge of ligands for ligpargen (when itp file should

be generated). If multiple ligands are present, give a

comma-delimited list.

-w WATERS, --waters WATERS

Water identifiers of crystalized water molecules

present within the PDB file.

-i IONS, --ions IONS Ion identifiers of crystalized ions present within the

PDB file.

--full_relax FULL_RELAX

Toggle for performing full MD relaxation. If set to

false, the run will finish after the initial

relaxation. (default = True)

--res RESTRAINT Position restraints during MD production run. Options:

bw (Ballesteros-Weinstein Restrained Relaxation -

default), ca (C-Alpha Restrained Relaxation)

-f LOOP_FILL, --loop_fill LOOP_FILL

Amount of Å per AA to fill cut loops. The total

distance is calculated from the coordinates of the

remaining residues. The AA contour length is 3.4-4.0

Å, To allow for flexibility in the loop, 2.0 Å/AA

(default) is suggested. (example: -f 2.0)

-q QUEUE, --queue QUEUE

Queueing system to use (slurm, pbs, pbs_ib and svgd

supported)

-d, --debug

SI 3. **Log.log file from PyMemDyn and snapshot of the corresponding memprot.Gpcr-ModSim interface showing the dynamically updated log file of the corresponding simulation**

2024-10-04,16:59:41 - pymemdyn - INFO - Pymemdyn started.

2024-10-04,16:59:41 - pymemdyn.protein.System - INFO - Initializing system

2024-10-04,16:59:41 - pymemdyn.protein.System - INFO - The PDB file contains the following cofactors: {'BNG', 'HOH', '6BG'}

2024-10-04,16:59:41 - pymemdyn.protein.System - INFO - The PDB file contains the following residues: {'ASP', 'PHE', 'THR', 'TRP', 'GLU', 'VAL', 'LYS', 'HIS', 'ARG', 'CYS', 'GLN', 'PRO', 'ILE', 'LEU', 'MET', 'ASN', 'GLY', 'SER', 'ALA', 'TYR'}

2024-10-04,16:59:41 - pymemdyn.protein.System - INFO - PDB file created containing protein(s): protein.pdb

2024-10-04,16:59:41 - pymemdyn.protein.System - INFO - PDB file created containing cofactor: 6BG.pdb

2024-10-04,16:59:41 - pymemdyn.checks.checkProtein - INFO - Identified chains: ['A']

2024-10-04,16:59:41 - pymemdyn.checks.checkProtein - INFO - Checking chain: A

2024-10-04,16:59:41 - pymemdyn.checks.checkProtein - INFO - No missing loops found

2024-10-04,16:59:41 - pymemdyn.checks.checkProtein - INFO - Residue 5: TYR atom counts (Counter({'C': 9, 'O': 2, 'N': 1})) does not match with reference atom counts (Counter({'C': 3, 'N': 1, 'O': 1})). It will be deleted from your pdb and replaced with MODELLER

2024-10-04,16:59:41 - pymemdyn.checks.checkProtein - INFO - Residue 187: ARG atom counts (Counter({'C': 6, 'N': 4, 'O': 1})) does not match with reference atom counts (Counter({'C': 3, 'N': 1, 'O': 1})). It will be deleted from your pdb and replaced with MODELLER

2024-10-04,16:59:41 - pymemdyn.checks.checkProtein - INFO - Residue 235: GLN atom counts (Counter({'C': 5, 'N': 2, 'O': 2})) does not match with reference atom counts (Counter({'C': 3, 'N': 1, 'O': 1})). It will be deleted from your pdb and replaced with MODELLER

2024-10-04,16:59:41 - pymemdyn.checks.checkProtein - INFO - Residue 305: LYS atom counts (Counter({'C': 6, 'N': 2, 'O': 1})) does not match with reference atom counts (Counter({'C': 3, 'N': 1, 'O': 1})). It will be deleted from your pdb and replaced with MODELLER

2024-10-04,16:59:41 - pymemdyn.checks.checkProtein - INFO - Residue 461: LYS atom counts (Counter({'C': 6, 'N': 2, 'O': 1})) does not match with reference atom counts (Counter({'C': 3, 'N': 1, 'O': 1})). It will be deleted from your pdb and replaced with MODELLER

2024-10-04,16:59:41 - pymemdyn.checks.checkProtein - INFO - Residue 479: GLU atom counts (Counter({'C': 5, 'O': 3, 'N': 1})) does not match with reference atom counts (Counter({'C': 3, 'N': 1, 'O': 1})). It will be deleted from your pdb and replaced with MODELLER

2024-10-04,16:59:41 - pymemdyn.protein.Monomer - INFO - Broken chains: ['A']

2024-10-04,16:59:52 - pymemdyn.run.Run - INFO - Center of protein at [ 0.61032414 -0.954552 2.99542014]

2024-10-04,16:59:52 - pymemdyn.protein.CalculateLigandParameters - INFO - initialization of CalculateLigandParameters started

2024-10-04,16:59:52 - pymemdyn.protein.CalculateLigandParameters - INFO - Ligand parameter file 6BG.ff not found.

2024-10-04,16:59:52 - pymemdyn.protein.CalculateLigandParameters - INFO - Ligand parameter file 6BG.itp not found.

2024-10-04,16:59:52 - pymemdyn.protein.CalculateLigandParameters - INFO - Ligand parameter files will be generated with Ligpargen.

2024-10-04,16:59:52 - pymemdyn.protein.CalculateLigandParameters - INFO - Molecule has explicit hydrogens: False

2024-10-04,16:59:52 - pymemdyn.protein.CalculateLigandParameters - INFO - Charge of 6BG was defined by the user as 0.

2024-10-04,16:59:52 - pymemdyn.protein.CalculateLigandParameters - WARNING - Hydrogens within Aromatic rings are likely to be incorrectly added. For compounds with Aromatic rings rings we recommend using explicit hydrogens.

2024-10-04,16:59:52 - pymemdyn.protein.CalculateLigandParameters - INFO - Adding hydrogens to 6BG.

2024-10-04,16:59:52 - pymemdyn.protein.CalculateLigandParameters - INFO - Calculating ligand parameters for 6BG using LigParGen. ifile: 6BG_lpg.pdb

2024-10-04,16:59:54 - pymemdyn.protein.CalculateLigandParameters - INFO - Calculated ligand parameters for 6BG using LigParGen.

2024-10-04,16:59:54 - pymemdyn.protein.CalculateLigandParameters - INFO - cofactors: ['6BG']

2024-10-04,16:59:54 - pymemdyn.protein.CalculateLigandParameters - INFO - Checking distance between 6BG and protein

2024-10-04,16:59:54 - pymemdyn.protein.CalculateLigandParameters - INFO - Distance between cofactor and protein center is 4.206006998196517

2024-10-04,16:59:54 - pymemdyn.protein.ProteinComplex - INFO - Initializing protein complex.

2024-10-04,16:59:54 - pymemdyn.run.Run - INFO -

[1/6]: Init

2024-10-04,16:59:54 - pymemdyn.gromacs.Gromacs - INFO - LigandInit Step (1/39): pdb2gmx.

2024-10-04,17:00:16 - pymemdyn.gromacs.Gromacs - INFO - LigandInit Step (2/39): set_itp.

2024-10-04,17:00:16 - pymemdyn.gromacs.Gromacs - INFO - LigandInit Step (3/39): clean_itp.

2024-10-04,17:00:16 - pymemdyn.gromacs.Gromacs - INFO - LigandInit Step (4/39): concat.

2024-10-04,17:00:16 - pymemdyn.gromacs.Gromacs - INFO - LigandInit Step (5/39): set_protein_height.

2024-10-04,17:00:16 - pymemdyn.gromacs.Gromacs - INFO - LigandInit Step (6/39): editconf.

2024-10-04,17:00:17 - pymemdyn.gromacs.Gromacs - INFO - LigandInit Step (7/39): set_protein_size.

2024-10-04,17:00:17 - pymemdyn.gromacs.Gromacs - INFO - LigandInit Step (8/39): editconf2.

2024-10-04,17:00:17 - pymemdyn.gromacs.Gromacs - INFO - LigandInit Step (9/39): set_protein_size2.

2024-10-04,17:00:17 - pymemdyn.gromacs.Gromacs - INFO - LigandInit Step (10/39): make_ndx_6BG.

2024-10-04,17:00:17 - pymemdyn.gromacs.Gromacs - INFO - LigandInit Step (11/39): genrestr_6BG.

2024-10-04,17:00:17 - pymemdyn.gromacs.Gromacs - INFO - LigandInit Step (12/39): set_popc.

2024-10-04,17:00:17 - pymemdyn.gromacs.Gromacs - INFO - LigandInit Step (13/39): editconf3.

2024-10-04,17:00:17 - pymemdyn.gromacs.Gromacs - INFO - LigandInit Step (14/39): editconf4.

2024-10-04,17:00:17 - pymemdyn.gromacs.Gromacs - INFO - LigandInit Step (15/39): make_topol.

2024-10-04,17:00:17 - pymemdyn.gromacs.Gromacs - INFO - LigandInit Step (16/39): editconf5.

2024-10-04,17:00:17 - pymemdyn.gromacs.Gromacs - INFO - LigandInit Step (17/39): solvate.

2024-10-04,17:00:17 - pymemdyn.gromacs.Gromacs - INFO - LigandInit Step (18/39): set_protein_height2.

2024-10-04,17:00:17 - pymemdyn.gromacs.Gromacs - INFO - LigandInit Step (19/39): set_water.

2024-10-04,17:00:18 - pymemdyn.gromacs.Gromacs - INFO - LigandInit Step (20/39): editconf6.

2024-10-04,17:00:18 - pymemdyn.gromacs.Gromacs - INFO - LigandInit Step (21/39): editconf7.

2024-10-04,17:00:18 - pymemdyn.gromacs.Gromacs - INFO - LigandInit Step (22/39): editconf8.

2024-10-04,17:00:18 - pymemdyn.gromacs.Gromacs - INFO - LigandInit Step (23/39): solvate2.

2024-10-04,17:00:20 - pymemdyn.gromacs.Gromacs - INFO - LigandInit Step (24/39): count_lipids.

2024-10-04,17:00:20 - pymemdyn.gromacs.Gromacs - INFO - LigandInit Step (25/39): make_topol2.

2024-10-04,17:00:20 - pymemdyn.gromacs.Gromacs - INFO - LigandInit Step (26/39): make_topol_lipids.

2024-10-04,17:00:20 - pymemdyn.gromacs.Gromacs - INFO - LigandInit Step (27/39): make_ffoplsaanb.

2024-10-04,17:00:20 - pymemdyn.gromacs.Gromacs - INFO - LigandInit Step (28/39): set_grompp.

2024-10-04,17:00:20 - pymemdyn.gromacs.Gromacs - INFO - LigandInit Step (29/39): set_chains.

2024-10-04,17:00:20 - pymemdyn.gromacs.Gromacs - INFO - LigandInit Step (30/39): make_ndx.

2024-10-04,17:00:21 - pymemdyn.gromacs.Gromacs - INFO - LigandInit Step (31/39): grompp.

2024-10-04,17:00:23 - pymemdyn.gromacs.Gromacs - INFO - LigandInit Step (32/39): trjconv.

2024-10-04,17:00:23 - pymemdyn.gromacs.Gromacs - INFO - LigandInit Step (33/39): get_charge.

2024-10-04,17:00:26 - pymemdyn.gromacs.Gromacs - INFO - LigandInit Step (34/39): genion.

2024-10-04,17:00:26 - pymemdyn.gromacs.Gromacs - INFO - LigandInit Step (35/39): grompp2.

2024-10-04,17:00:28 - pymemdyn.gromacs.Gromacs - INFO - LigandInit Step (36/39): trjconv2.

2024-10-04,17:00:29 - pymemdyn.gromacs.Gromacs - INFO - LigandInit Step (37/39): grompp3.

2024-10-04,17:00:31 - pymemdyn.gromacs.Gromacs - INFO - LigandInit Step (38/39): trjconv3.

2024-10-04,17:00:32 - pymemdyn.gromacs.Gromacs - INFO - LigandInit Step (39/39): clean_pdb.

2024-10-04,17:00:32 - pymemdyn.run.Run - INFO -

[2/6]: Minimization

2024-10-04,17:00:32 - pymemdyn.gromacs.Gromacs - INFO - BasicMinimization Step (1/2): set_stage_init.

2024-10-04,17:00:32 - pymemdyn.gromacs.Gromacs - INFO - BasicMinimization Step (2/2): mdrun.

2024-10-04,17:01:52 - pymemdyn.run.Run - INFO -

[3/6]: Equilibration

2024-10-04,17:01:52 - pymemdyn.gromacs.Gromacs - INFO - BasicEquilibration Step (1/8): clean_itp.

2024-10-04,17:01:52 - pymemdyn.gromacs.Gromacs - INFO - BasicEquilibration Step (2/8): editconf.

2024-10-04,17:01:52 - pymemdyn.gromacs.Gromacs - INFO - BasicEquilibration Step (3/8): make_ndx.

2024-10-04,17:01:53 - pymemdyn.gromacs.Gromacs - INFO - BasicEquilibration Step (4/8): set_grompp.

2024-10-04,17:01:54 - pymemdyn.gromacs.Gromacs - INFO - BasicEquilibration Step (5/8): set_stage_init.

2024-10-04,17:01:54 - pymemdyn.gromacs.Gromacs - INFO - BasicEquilibration Step (6/8): grompp.

2024-10-04,17:01:56 - pymemdyn.gromacs.Gromacs - INFO - BasicEquilibration Step (7/8): set_stage_init2.

2024-10-04,17:01:56 - pymemdyn.gromacs.Gromacs - INFO - BasicEquilibration Step (8/8): mdrun.

2024-10-04,17:50:01 - pymemdyn.run.Run - INFO -

[4/6]: Relax

2024-10-04,17:50:01 - pymemdyn.gromacs.Gromacs - INFO - BasicRelax Step (1/16): relax800.

2024-10-04,17:50:01 - pymemdyn.gromacs.Gromacs - INFO - BasicRelax Step (2/16): set_stage_init800.

2024-10-04,17:50:01 - pymemdyn.gromacs.Gromacs - INFO - BasicRelax Step (3/16): grompp800.

2024-10-04,17:50:04 - pymemdyn.gromacs.Gromacs - INFO - BasicRelax Step (4/16): mdrun800.

2024-10-04,18:37:55 - pymemdyn.gromacs.Gromacs - INFO - BasicRelax Step (5/16): relax600.

2024-10-04,18:37:55 - pymemdyn.gromacs.Gromacs - INFO - BasicRelax Step (6/16): set_stage_init600.

2024-10-04,18:37:55 - pymemdyn.gromacs.Gromacs - INFO - BasicRelax Step (7/16): grompp600.

2024-10-04,18:37:58 - pymemdyn.gromacs.Gromacs - INFO - BasicRelax Step (8/16): mdrun600.

2024-10-04,19:25:45 - pymemdyn.gromacs.Gromacs - INFO - BasicRelax Step (9/16): relax400.

2024-10-04,19:25:45 - pymemdyn.gromacs.Gromacs - INFO - BasicRelax Step (10/16): set_stage_init400.

2024-10-04,19:25:45 - pymemdyn.gromacs.Gromacs - INFO - BasicRelax Step (11/16): grompp400.

2024-10-04,19:25:48 - pymemdyn.gromacs.Gromacs - INFO - BasicRelax Step (12/16): mdrun400.

2024-10-04,20:13:41 - pymemdyn.gromacs.Gromacs - INFO - BasicRelax Step (13/16): relax200.

2024-10-04,20:13:41 - pymemdyn.gromacs.Gromacs - INFO - BasicRelax Step (14/16): set_stage_init200.

2024-10-04,20:13:41 - pymemdyn.gromacs.Gromacs - INFO - BasicRelax Step (15/16): grompp200.

2024-10-04,20:13:44 - pymemdyn.gromacs.Gromacs - INFO - BasicRelax Step (16/16): mdrun200.

2024-10-04,21:01:39 - pymemdyn.run.Run - INFO -

[5/6]: CARelax

2024-10-04,21:01:39 - pymemdyn.gromacs.Gromacs - INFO - BasicCARelax Step (1/5): set_stage_init.

2024-10-04,21:01:40 - pymemdyn.gromacs.Gromacs - INFO - BasicCARelax Step (2/5): set_stage_init2.

2024-10-04,21:01:40 - pymemdyn.gromacs.Gromacs - INFO - BasicCARelax Step (3/5): restrain_ca.

2024-10-04,21:01:40 - pymemdyn.gromacs.Gromacs - INFO - BasicCARelax Step (4/5): grompp.

2024-10-04,21:01:43 - pymemdyn.gromacs.Gromacs - INFO - BasicCARelax Step (5/5): mdrun.

2024-10-05,01:00:59 - pymemdyn.run.Run - INFO -

[6/6]: CACollectResults

2024-10-05,01:00:59 - pymemdyn.gromacs.Gromacs - INFO - BasicCACollectResults Step (1/20): trjcat.

2024-10-05,01:01:00 - pymemdyn.gromacs.Gromacs - INFO - BasicCACollectResults Step (2/20): trjconv.

2024-10-05,01:01:02 - pymemdyn.gromacs.Gromacs - INFO - BasicCACollectResults Step (3/20): eneconv.

2024-10-05,01:01:02 - pymemdyn.gromacs.Gromacs - INFO - BasicCACollectResults Step (4/20): rms1.

2024-10-05,01:01:04 - pymemdyn.gromacs.Gromacs - INFO - BasicCACollectResults Step (5/20): rms2.

2024-10-05,01:01:05 - pymemdyn.gromacs.Gromacs - INFO - BasicCACollectResults Step (6/20): rms3.

2024-10-05,01:01:06 - pymemdyn.gromacs.Gromacs - INFO - BasicCACollectResults Step (7/20): rmsf.

2024-10-05,01:01:08 - pymemdyn.gromacs.Gromacs - INFO - BasicCACollectResults Step (8/20): tot_ener.

2024-10-05,01:01:08 - pymemdyn.gromacs.Gromacs - INFO - BasicCACollectResults Step (9/20): temp.

2024-10-05,01:01:08 - pymemdyn.gromacs.Gromacs - INFO - BasicCACollectResults Step (10/20): pressure.

2024-10-05,01:01:08 - pymemdyn.gromacs.Gromacs - INFO - BasicCACollectResults Step (11/20): volume.

2024-10-05,01:01:08 - pymemdyn.gromacs.Gromacs - INFO - BasicCACollectResults Step (12/20): set_stage_init.

2024-10-05,01:01:08 - pymemdyn.gromacs.Gromacs - INFO - BasicCACollectResults Step (13/20): grompp.

2024-10-05,01:01:10 - pymemdyn.gromacs.Gromacs - INFO - BasicCACollectResults Step (14/20): set_end.

2024-10-05,01:01:10 - pymemdyn.gromacs.Gromacs - INFO - BasicCACollectResults Step (15/20): set_end_2.


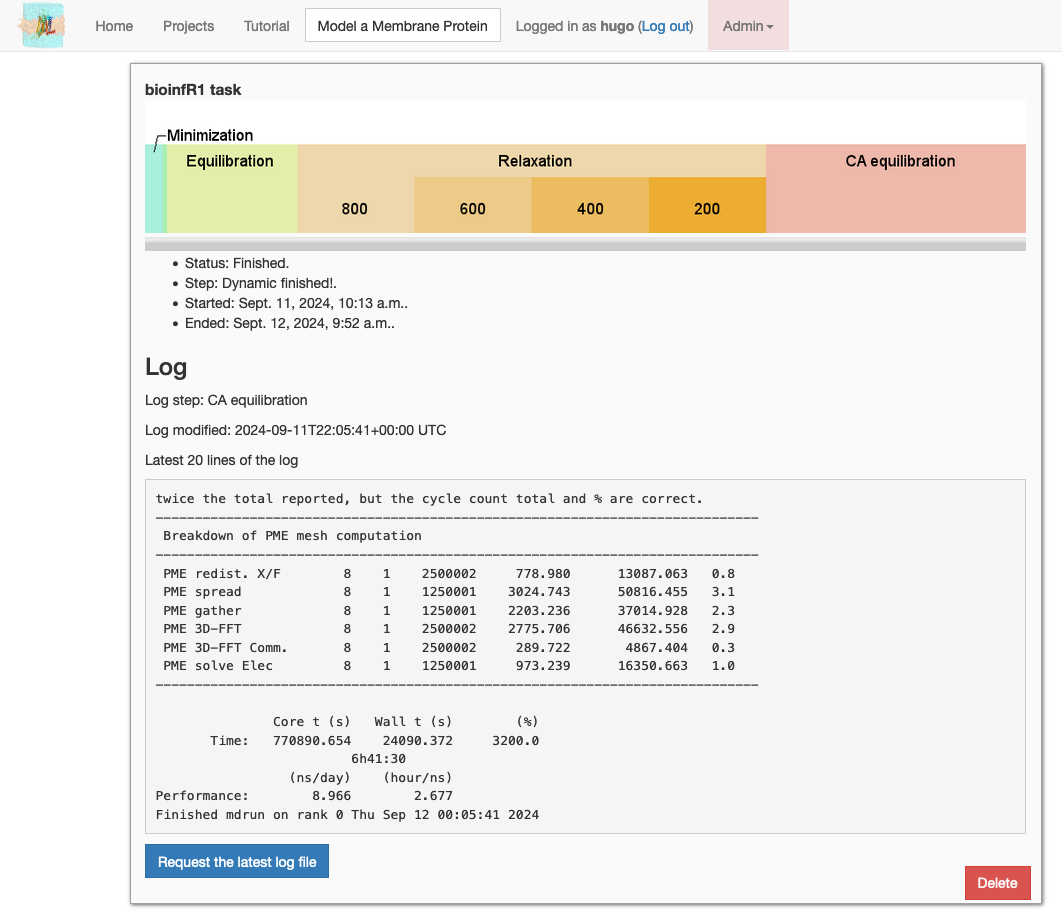


SI 4. **Embedded and equilibrated structures by Memprot.GPCR-ModSim**

**
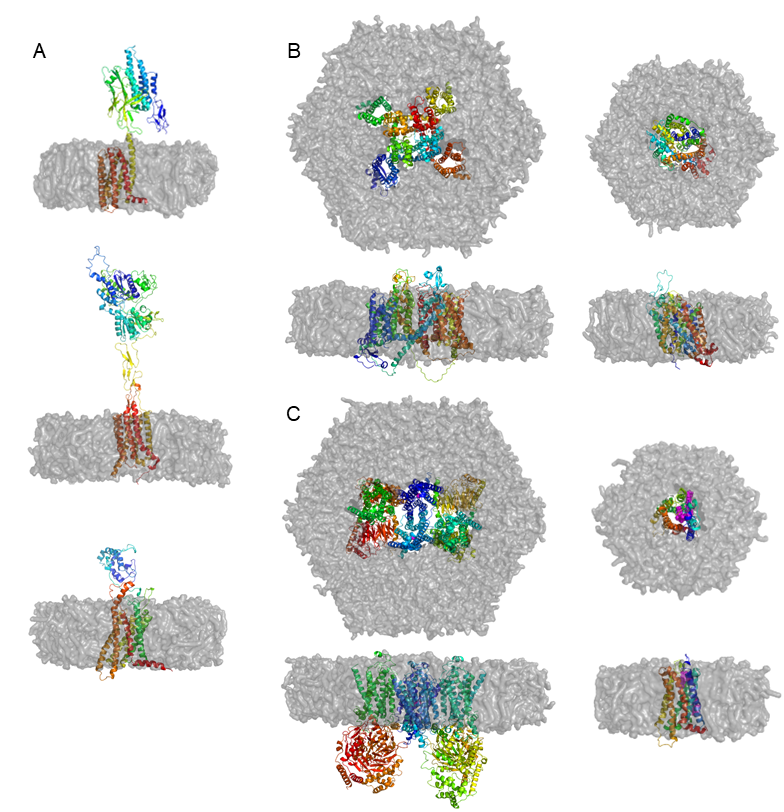
**

Target variety using the PyModSim and PyMemDyn modules. (**A**) Embedded homology models of GPCRs that do not have an experimentally determined structure. These proteins include the class B2 GPCR L_1_ (top), the class C GPCR mGlu_6_ (center), and the class F GPCR frizzled-6 (bottom). (**B)** This is also expanded to other protein superfamilies, as demonstrated with the ion channel Na channel protein type 11 subunit alpha (left), and the SLC DAT_1_ (right). (**C**) Embedded protein complexes simulated by PyMemDyn. These complexes include the A_1_-A_2A_ heteromer with two bound G proteins (left) and CB_1_ (PDB: 6KQI) with an orthosteric and allosteric ligand in magenta (right).
